# Two new species of benthopelagic *Stephos* (Copepoda, Calanoida, Stephidae) from Korea

**DOI:** 10.3897/zookeys.495.7862

**Published:** 2015-04-08

**Authors:** Seong Yong Moon, Seok-Hyun Youn, B. A. Venmathi Maran

**Affiliations:** 1 Fisheries and Ocean Information Division, National Fisheries Research & Development Institute, Busan 619-705, Korea; 2 Marine Ecosystem Research Division, Korea Institute of Ocean Science & Technology, P. O., Box 29, Ansan, Seoul 425-600, Korea

**Keywords:** *Stephos*, new species, benthopelagic, intertidal mud-flat, commensal, Korea

## Abstract

Two new species of benthopelagic copepods of the genus *Stephos* T. Scott, 1892, belonging to the family Stephidae G.O. Sars, 1902, are described based on specimens collected in the stagnant water flooding the burrows excavated by ocypodid crabs in two intertidal mud-flats, and from near-bottom shallow waters in Korea, respectively. They can be easily diagnosed based on the ornamentation of both the female genital double-somite and genital operculum; the morphology of the distal segment of the male right P5; the presence/absence of a tiny pointed process on the distomedial angle of second segment of female P5; and the condition (seta or spine) of the lateral armature element on the distal segment of female fifth legs, among other features. This is one of the few cases reported of calanoid copepods living as commensals of other invertebrates, and raises to six the number of members of the genus reported from Asia. This is also the first record of the family Stephidae in Korea.

## Introduction

The benthopelagic calanoid family Stephidae G.O. Sars, 1902, consists of four valid genera: *Miostephos* Bowman, 1976, *Parastephos* G.O. Sars, 1902, *Speleohvarella* Kršinić, 2005, and *Stephos* T. Scott, 1892 (Boxshall and Halsey 2004; Kršinić 2005). The genus *Stephos* is the largest and more primitive, currently comprising 28 valid species found in anchialine and marine coastal near-bottom habitats from tropical to polar regions (Ohtsuka and Hiromi 1987; Bradford-Grieve 1999; Zagami et al. 2000; Boxshall and Halsey 2004; Jaume et al. 2008; Kršinić 2012). They are mostly benthopelagic and can be collected with dredges and sledges (Fosshagen 1970; Ohtsuka and Hiromi 1987; Bradford-Grieve 1999; Zagami et al. 2000). Most of them have so far been recorded from the North Atlantic and adjacent waters (Boxshall and Halsey 2004; Jaume et al. 2008; Kršinić 2012), and also from the Indo-Pacific region (Mori 1942; Chen and Zhang 1965; Andronov 1974; Bradford-Grieve 1999; Ohtsuka and Hiromi 1987).

In Korean waters, only three species of benthopelagic calanoids have so far been reported, namely: *Sarsarietellus orientalis* Soh et al., 2013 (Arietellidae), collected from the shallow water near-bottom of the Jeju Island, southern Korea (Soh et al. 2013), and *Paramisophria sinjinensis* Lim & Min, 2014 and *Paramisophria koreana* Lim & Min, 2014 (Arietellidae), both collected also from near-bottom shallow waters in southern Korea (Lim and Min 2014). The recent advances in the knowledge of species diversity of benthopelagic calanoids in the region is the result of intensive investigations using a diverse array of sampling methods.

During the general field surveys carried out recently to collect calanoid copepods from two inter-tidal mud flats and near-bottom shallow waters, two new species of the genus *Stephos* were recorded. This paper deals with their descriptions and presents the first record of the family in Korean waters.

## Material and methods

Copepods were collected from the stagnant water retained in the burrows excavated by ocypodid crabs in two intertidal mud-flats using a hand net (0.2 mm mesh size) and also from near-bottom shallow waters using a light trap and a plankton net (0.2 mm mesh size) at high tide at dusk hours in eastern and southern Korea. For morphological examination, samples were fixed in 5% natural formalin-seawater solution. Specimens were later cleared in 70% lactic acid for 1 to 2 hours before dissecting under the dissection microscope (Nikon) in a drop of lactophenol on a wooden slide (Humes and Gooding 1964). The removed body parts and appendages were examined under a Olympus BX51 phase contrast microscope up to ×1,000. Drawings were made with the aid of a drawing tube attached to the microscope.

Body sizes of individuals were measured using a stage micrometer from the head to the tip of the caudal rami excluding caudal setae. The morphological terminology follows Huys and Boxshall (1991). Abbreviations used in the text and figures are as follows: ae, aesthetasc; P1-P5, first to fifth swimming legs. Specimens are deposited at the National Institute of Biological Resources (NIBR), Incheon, Korea.

## Results

### Order Calanoida G.O. Sars, 1903 Family Stephidae G.O. Sars, 1902 Genus *Stephos* T. Scott, 1892

#### Stephos geojinensis sp. n.

Taxon classificationAnimaliaCalanoidaStephidae

http://zoobank.org/57A20501-74CF-4E13-874D-4FC21A3C7ECE

Figs 1 , 2 , 3 , 4

##### Material examined.

Female holotype (NIBRIV0000304586) and male allotype (NIBRIV0000304587) undissected and preserved in 70% ethanol; female paratype (NIBRIV0000304738) and male paratype (NIBRIV0000304739) dissected on two glass slides; one female paratype and seven male paratypes (NIBRIV0000304293, 1 vial) preserved in 70% ethanol. All specimens were collected from the near-bottom using a light trap at high tide at dusk, on 28 August 2010 by the senior author (S. Y. Moon). The description below is based on the paratypes.

##### Type locality.

Geojin fishery port, Gosung-gun, Gangwon-do (approximately 38°26'58"N 128°27'46"E), Korea.

##### Female.

**Figure 1.**
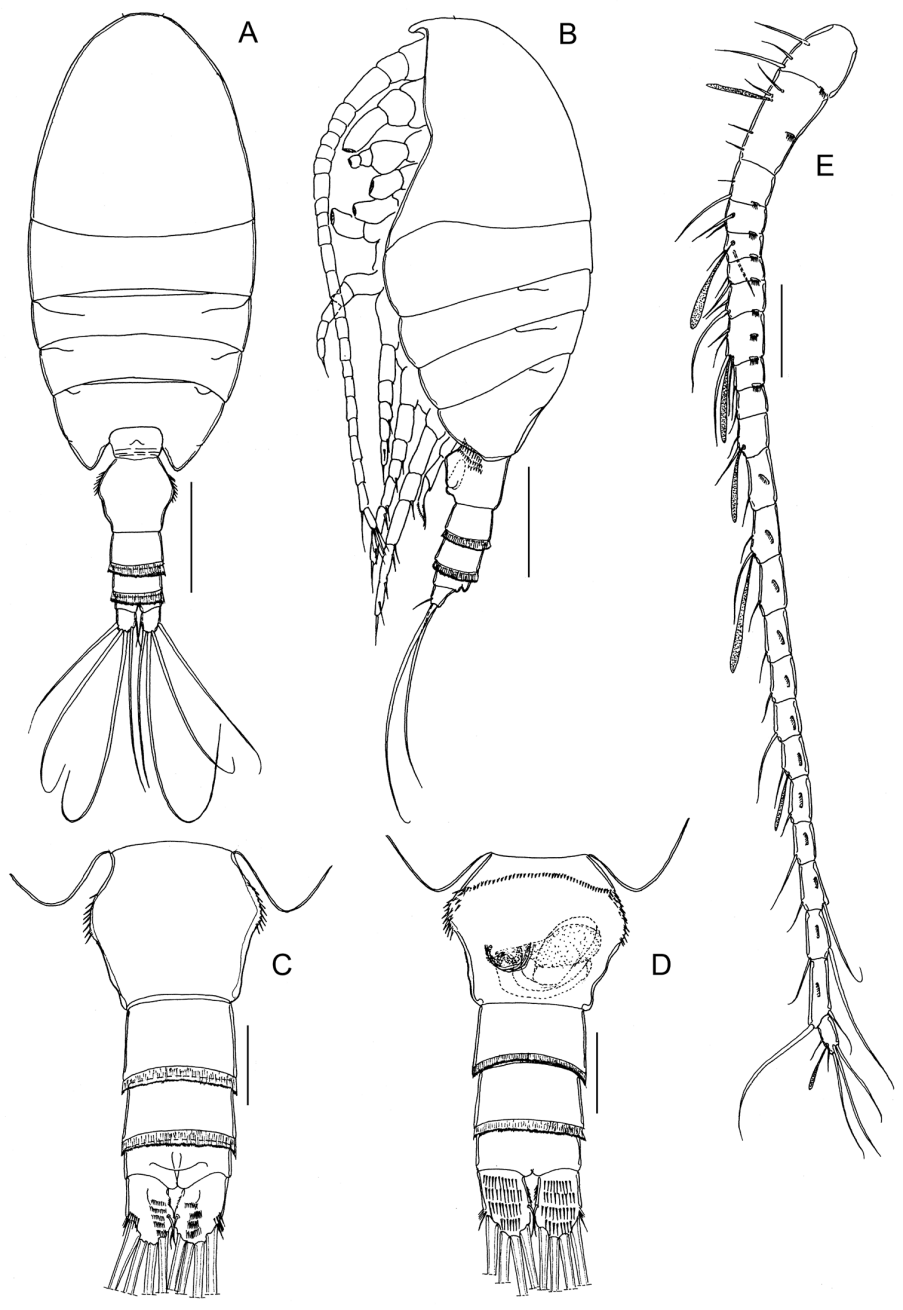
*Stephos geojinensis* sp. n., female paratype. **A** habitus, dorsal view **B** habitus, lateral view **C** urosome, dorsal view **D** urosome and genital double-somite, ventral view **E** antennule. Scale bars: **A, B** = 200 µm; **C–E** = 50 µm.

Body (Fig. 1A, B) robust, length 883 μm. Prosome 5-segmented; cephalosome and first pedigerous somites completely separated; fourth and fifth pedigerous somites completely fused, posterior corners of prosome symmetrical. Rostrum represented by rounded knob. Prosome-urosome ratio 2.42:1. Urosome (Fig. 1C, D) 4-segmented, comprising genital double-somite, two free abdominal somites and anal somite; length ratio of urosome somites as 48.7: 17.0: 19.8: 14.5 = 100. Genital double-somite (Fig. 1C, D) symmetrical with proximolateral margins produced in dorsal aspect (Fig. 1C), asymmetrical in ventral aspect with smooth evenly rounded operculum displaced to the right; double-somite with row of spinules anteriorly on ventral surface and patch of spinules at each side as figured. First and second free abdominal somites with transverse hyaline frill both dorsally and ventrally. Anal somite (Fig. 1C, D) shortest. Caudal rami (Fig. 1C, D), with 6 setae, symmetrical, about 1.35 times longer than wide (43 × 31 μm), with several rows of spinules on dorsal and ventral surface; caudal seta I absent; seta II reduced; seta VII displaced to medial margin.

Antennules (Fig. 1E) symmetrical, extending beyond distal margin of second urosomite; 24-segmented, with ancestral segments I-II, III-IV, X-XI, and XXVII-XXVIII fused. Segmentation and setation pattern as follows (ancestral segment number-setae+aesthetasc): I-II-3s; III-IV-4s + 1ae, V-2s, VI-2s, VII-2s + 1ae, VIII-2s, IX-2s, X-XI-4s + 1ae, XII-1s, XIII-1s, XIV-2s + 1ae, XV-1s, XVI-2s + 1ae, XVII-1s, XVIII-1s, XIX-1s, XX-1s, XXI-1s + 1ae, XXII-1s, XXIII-1s, XXIV-1s +1s, XXV–1s +1s, XXVI–1s +1s, XXVII-XXVIII–5s + 1ae. Ancestral segments II, III, V-XII, and XV-XXVI each with row of spinules on posterior surface.

**Figure 2.**
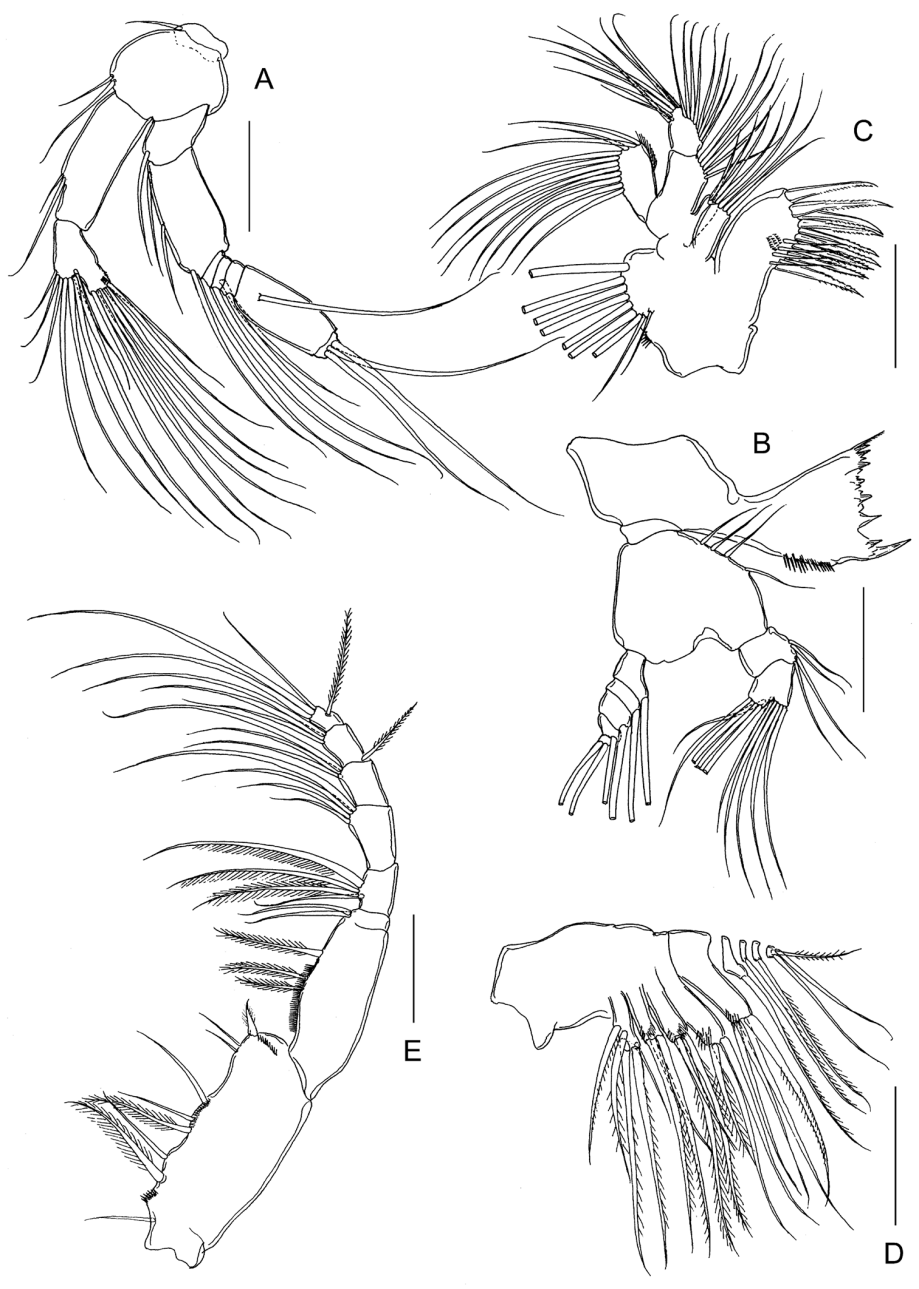
*Stephos geojinensis* sp. n., female paratype. **A** antenna **B** mandible **C** maxillule **D** maxilla **E** maxilliped. Scale bars = 50 µm.

Antenna (Fig. 2A) biramous; coxa and basis separate, bearing 1 and 2 setae on distomedial angle, respectively; endopod 2-segmented, proximal segment with 2 setae, compound distal segment bilobed with 8 and 7 setae, respectively, outer margin with small serrated process subdistally and tiny spinule adjacent to serrated process; exopod 7-segmented, setal formula 1, 3, 1, 2, 1, 1, 3.

Mandible (Fig. 2B) with well developed coxal gnathobase, cutting edge with isolated unicuspid tooth and 7 heterogeneous teeth plus dorsal spinulose seta. Mandibular palp biramous; basis with 4 setae on inner margin. Exopod 5-segmented, setal formula 1, 1, 1, 1, 2; endopod 2-segmented, proximal segment with 4 setae, distal segmentwith 10 setae.

Maxillule (Fig. 2C) praecoxa and coxa incompletely fused; praecoxal arthrite with 9 marginal spines plus 4 stiff setae on posterior surface; several rows of tiny spinules on posterior surface as figured. Coxal epipodite with 9 setae; coxal endite with 3 stiff setae. Proximal basal endite with 4 setae; distal basal endite indistinct, with 5 setae; no trace of basal exite. Exopod with 11 marginal setae; row of setules along distal portion of medial margin. Endopod apparently unsegmented with 4, 4, 7 setae.

Maxilla (Fig. 2D), indistinctly 6-segmented. Armature of praecoxal and coxal endites as 5, 3, 3, 3, respectively. Basal endite with 4 setae, 1 stouter than rest. Endopod 4-segmented, setal formula 1, 1, 1, 3. Praecoxal, coxal and basal endites with cluster of long spinules subdistally on lateral surface.

Maxilliped (Fig. 2E) syncoxa robust, with setal formula 1, 2, 3, 3 and several oblique rows of tiny spinules as figured; basis with 3 setae and row of setules on medial margin; endopod 6-segmented with proximal segment partially incorporated into basis, setal formula 2, 4, 4, 3, 3+1, 4.

**Figure 3.**
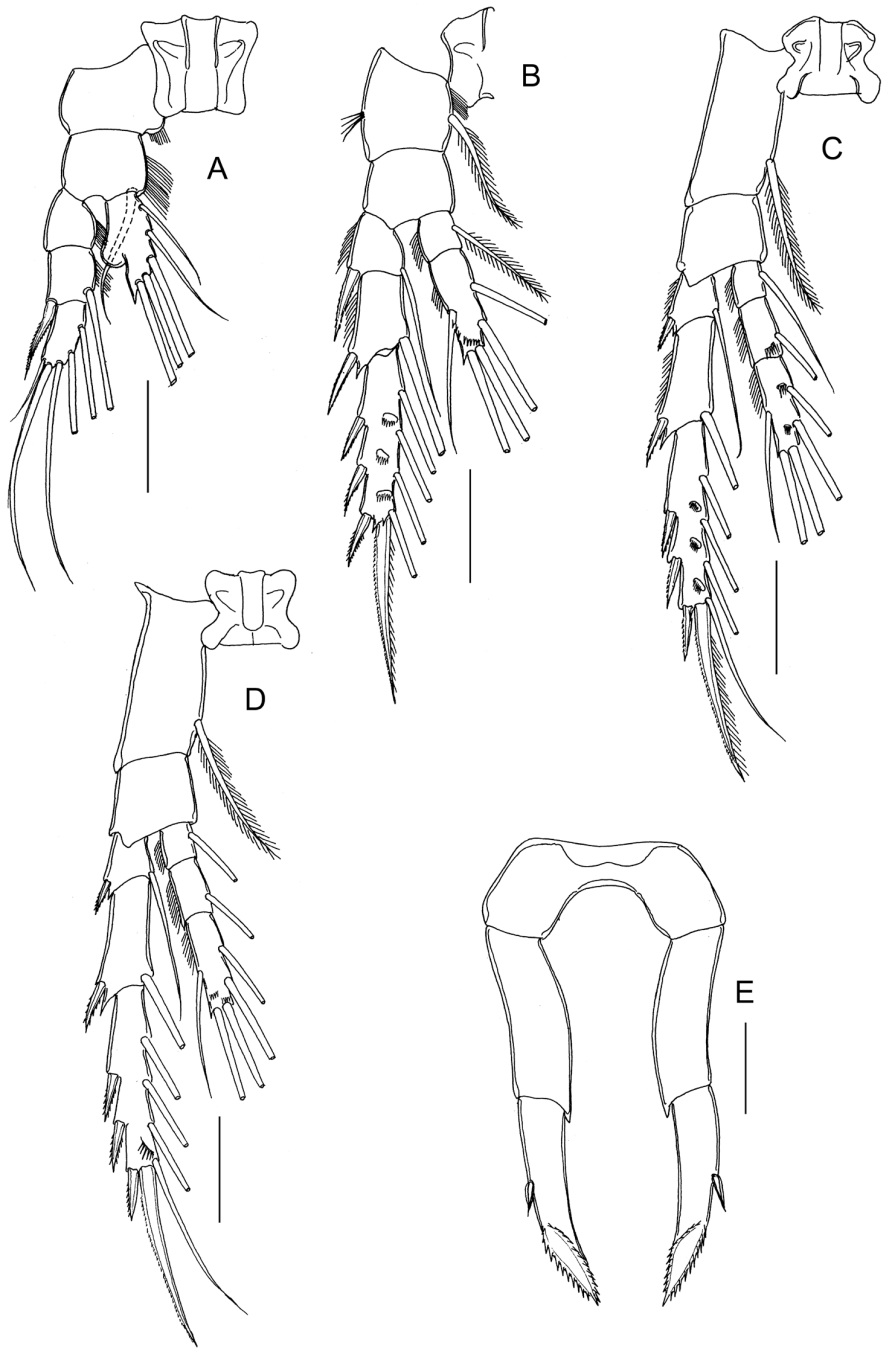
*Stephos geojinensis* sp. n., female paratype. **A** P1 **B** P2 **C** P3 **D** P4 **E** fifth legs. Scale bars = 50 µm.

**Figure 4.**
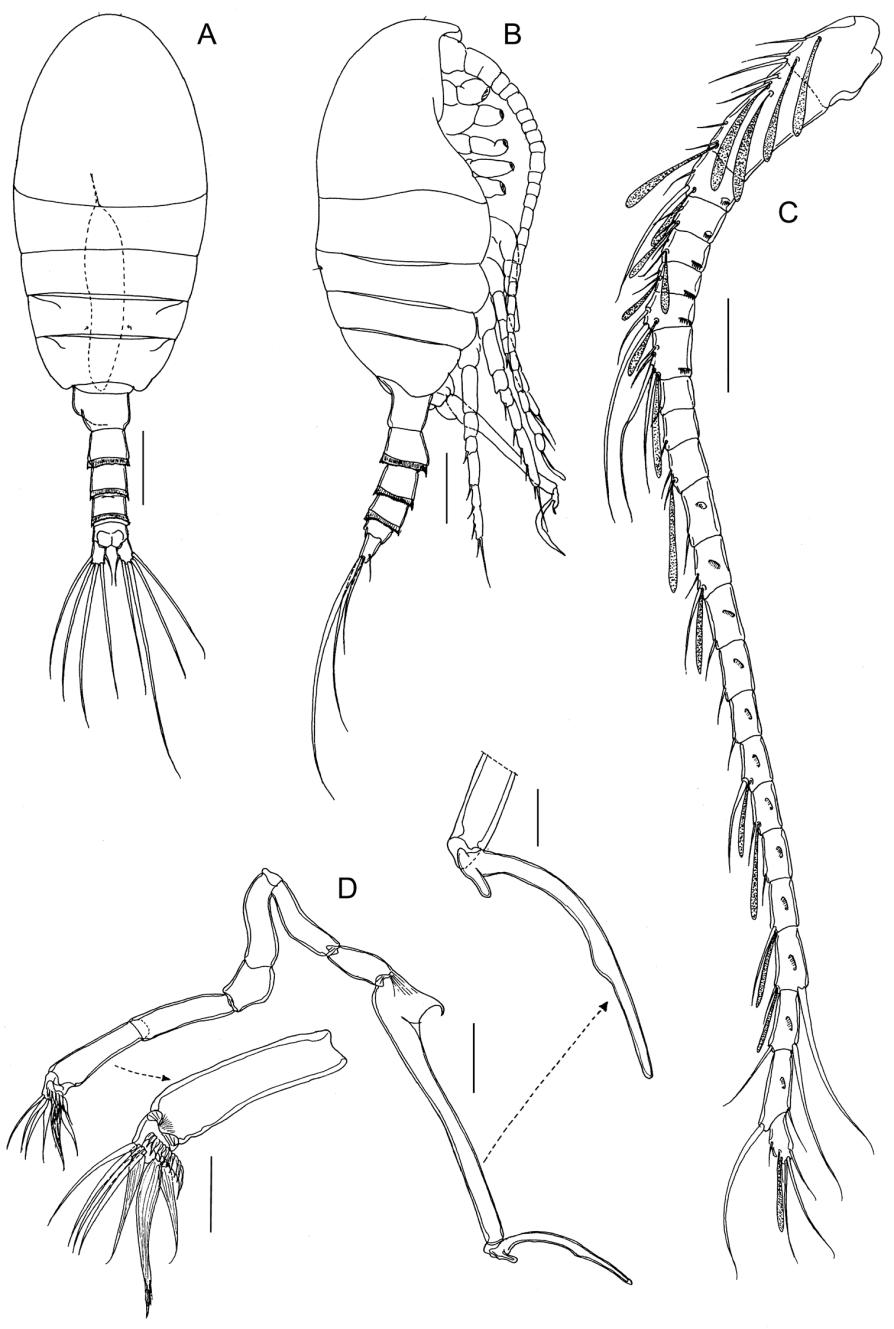
*Stephos geojinensis* sp. n., male paratype. **A** habitus, dorsal view **B** habitus, lateral view **C** antennule **D** fifth legs. Scale bars: **A, B** = 200 µm; **C, D** = 50 µm.

P1 to P4 (Fig. 3A–D) progressively larger towards posterior, each comprising coxa, basis and 3-segmented exopod; endopod of P1 (Fig. 3A) 1-segmented, that of P2 (Fig. 4B) 2-segmented; endopods of P3 (Fig. 3C) and P4 (Fig. 3D) 3-segmented. Endopod of P2-P4 with transverse row of spinules distally on terminal segment. Exopod of P2-P4 with row of spinules on anterior surface of terminal segment. Armature formula of P1-P4 as follows (Roman numerals indicate spines, Arabic numeral indicate setae):

**Table:** Armature formula of P1-P4.

Legs	Coxa	Basis	Exopod	Endopod
P1	0-0	0-1	0-0; I-1; 1,2,2	0,2,3
P2	0-1	0-0	I-1; I-1; III,I,4	0-1; 1,2,2
P3	0-1	0-0	I-1; I-1; III,I,4	0-1; 0-1; 1,2,2
P4	0-1	0-0	I-1; I-1; III,I,4	0-1; 0-1; 1,2,2

Fifth legs (Fig. 3E) symmetrical, uniramous, 3-segmented with proximal segment fused to intercoxal sclerite; second segment elongated, 2.62 times longer than wide (42 × 16 μm), with distomedial angle produced into tiny pointed process. Distal segment elongated, tapering with short spine implanted mid-laterally and coarsely serrated spine incorporated (i.e. non-articulating) to segment distally.

##### Male.

Body (Fig. 4A, B) robust, length 819 μm (mean 821±0.06, n=4). Prosome 5-segmented; cephalosome and first pedigerous somite almost completely separated; fourth and fifth pedigerous somites completely fused; fifth pedigerous somite symmetrical, with lateral lobe at each side. Rostrum as in female. Prosome-urosome ratio 2.12:1. Urosome 5-segmented, comprising genital somite, three free abdominal somites and anal somite; length ratio of urosomites as 28.3: 20.5: 18.6: 17.0: 15.7 = 100. Genital somite asymmetrical, with lobe protruding anterolaterally on left side. First to third abdominal somites with transverse hyaline frill both dorsally and ventrally. Anal somite shortest. Caudal rami similar to those of the female.

Antennules (Fig. 4C) extending beyond distal margin of second urosomite, non-geniculate, 24-segmented with failure to express articulations between ancestral segments I-IV (although vestige of articulation between segments II and III expressed dorsally), X-XI and XXVII-XXVIII. Segmentation and setation pattern as follows (ancestral segment number-setae+aesthetasc): I-II – 3s + 2ae, III-IV– 4s +3ae, V–2s + 1ae, VI–2s+1ae, VII–2s + 1ae, VIII–2s+1ae, IX–2s + 1ae, X-XI–4s + 1ae, XII–1s, XIII–1s, XIV–2s + 1ae, XV–1s, XVI–2s + 1ae, XVII–1s, XVIII–1s, XIX–1s, XX–1s + 1ae, XXI–1s + 1ae, XXII–1s, XXIII–1s + 1ae, XXIV–1s +1s + 1ae, XXV–1s +1s, XXVI–1s +1s, XXVII-XXVIII–5s+ 1ae. Ancestral segments V-IX, XI and XV-XXVI each with row of spinules on posterior surface.

Antenna, mandible, maxillule, maxilla, maxilliped and P1-P4 similar to those of female. Fifth legs (Fig. 4D) strongly asymmetrical, uniramous and filiform. Left leg 5-segmented, shorter than right counterpart; second segment with blunt prominence medially; third and fourth segments elongated, about equal in length; distal segment reduced, with row of seven unequal long and 13 short hyaline lamellae disposed as figured. Right leg 4-segmented; third segment very elongated with short, curved proximolateral spur-like process; distal segment elongated and curved, bifid with short inner branch.

##### Etymology.

The specific name *geojinensis* is taken after the type locality Geojin Port, Gosung-gun, Gangwon-do, Korea.

##### Remarks.

*Stephos geojinensis* sp. n. is easily recognizable by the display of the following five diagnostic features: (1) female genital double-somite with protruding proximolateral margins in dorsal aspect; (2) genital double-somite with row of spinules anteriorly on ventral surface and patch of spinules at each side; (3) basis and distal segment of P5 elongated in female; (4) distal segment tapering with short spine implanted mid-laterally and coarsely serrated spine incorporated to segment distally in female P5; and (5) male right P5 distal segment elongated and curved, bifid with short inner branch.

Bradford-Grieve (1999) divided the species of *Stephos* in four groups based on the morphology of the male fifth legs. *Stephos geojinensis* falls within a “group IV” characterized by a 4-segmented male right P5 combined with a left leg with a narrow fourth segment. This group includes eight species from the western Pacific and the Atlantic: *Stephos angulatus* Bradford-Grieve, 1999, *Stephos marsalensis* Costanzo, Campolmi & Zagami, 2000, *Stephos morii* Greenwood, 1978, *Stephos pacificus* Ohtsuka & Hiromi, 1987, *Stephos pentacanthos* Chen & Zhang, 1965, *Stephos rustadi* Strömgren, 1969, *Stephos tsuyazakiensis* Tanaka, 1967, and *Stephos vivesi* Jaume, Boxshall & Gràcia, 2008 (see Table 1 in Bradford-Grieve 1999; Costanzo et al. 2000; Jaume et al. 2008).

The male fifth legs are diagnostic to distinguish *Stephos goejinensis* from other congeners in this group. Thus, *Stephos angulatus* is easily differentiated from the new species by the more developed inner branch of thebifid distal segment of the right P5, and by the distal segment of the left male P5 with only three elongate hyaline lamellae and a rounded cluster of short spinules (see Bradford-Grieve 1999). In *Stephos marsalensis*, the distal segment of right male P5 is not bifid whereas there are only 5 lamellate hyaline processes on the distal segment of left male P5 (see Costanzo et al. 2000).

*Stephos morii* differs from the new species in having the right P5 pseudochelate with a large inner branch on the distal segment,and the left leg carrying about 5 lamellate processes on the distal segment, which is produced into a long spinous process about 1.6 times longer than the segment (see Greenwood 1978 as *Stephos tropicus*). In *Stephos pacificus*, the distal segment of the right leg is not bifid and is bordered by a narrow lamella, whereas the left leg carries three terminal and two subterminal lamellate processes on the distal segment (see Ohtsuka and Hiromi 1987).

*Stephos rustadi* is easily separated from the new species by having the segment 3 of the right leg slightly shorter than segment 4, which terminates in a finely serrated claw-like structure, whereas the left leg carries two strong hook-like processes on the terminal segment, the larger one bifid (see Strömgren 1969).

*Stephos pentacanthos* and *Stephos tsuyazakiensis* share with the new species the same ornamentation on the male P5, but the new species has 7 unequal long and 13 short hyaline lamellae on the distal segment of left leg and a bifid distal segment with a short inner branch on right leg (Chen and Zhang 1965; Tanaka 1966).

Finally, *Stephos vivesi* can be differentiated from *Stephos goejinensis* based on the male right fifth leg distal segment, which is spatulate and displays two rounded outgrowths proximally on the anterior surface (vs. segment not spatulate, slender and bifid in *Stephos goejinensis*) (see Jaume et al. 2008).

#### Stephos projectus sp. n.

Taxon classificationAnimaliaCalanoidaStephidae

http://zoobank.org/9B90D397-EE66-4873-89C2-18D5AA6EC35F

Figs 5 , 6 , 7 , 8

##### Material examined.

Female holotype (NIBRIV0000304294) and male allotype (NIBRIV0000304297) undissected and preserved in 70% ethanol; 20 female paratypes (NIBRIV0000304295), and four male paratypes (NIBRIV0000304296) preserved in 70% ethanol. Dissected paratypes of both sexes are retained in the collection of the senior author. All specimens were collected at the type locality using a hand net on 28 February 2013 by the senior author (S. Y. Moon). The description below is based on the paratypes.

##### Additional material.

Female (NIBRIV0000304584) undissected, preserved in ethanol, female (NIBRIV0000304585) dissected on 1 glass slide, Daeyari, Wando Island, Wando-gun, Jeollanam-do, Korea, 19 July 2010.

##### Type locality.

Stagnant water in burrows of ocypodid crabs in intertidal mud flat, Bongyoungri, Naro Island, Goheung-gun, Jeollanam-do (approximately 34°29'13"N, 127°29'12"E), Korea.

##### Etymology.

The specific name, *projectus*, is derived from the dorsolateral spiniform projections present on the female genital double-somite.

##### Female.

**Figure 5.**
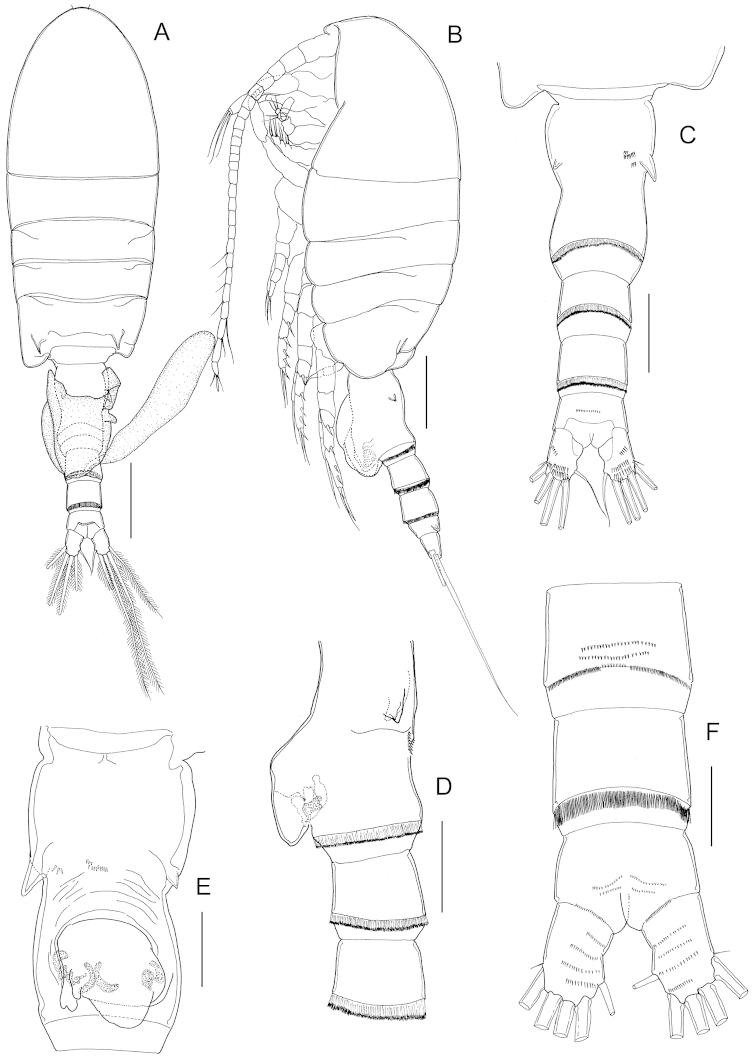
*Stephos projectus* sp. n., female paratype. **A** habitus, dorsal view **B** habitus, lateral view **C** fifth pedigerous somite and urosome, dorsal view **D** urosome, lateral view **E** genital double-somite, ventral view **F** second urosomal somite to caudal rami, ventral view. Scale bars: **A, B** = 200 μm; **C, D** = 100 μm; **E, F** = 50 μm.

Body (Fig. 5A, B) robust, length 1.51 mm (mean 1.54±0.07, *n* = 5). Prosome 5-segmented; cephalosome and first pedigerous somite completely separated; fourth and fifth pedigerous somites incompletely fused, posterior corners of fifth pedigerous somite slightly asymmetrical. Rostrum represented by rounded knob. Prosome-urosome ratio 1.83:1. Urosome 4-segmented (Fig. 5C, D), comprising genital double-somite, two free abdominal somites and anal somite; length ratio of urosomites as 51.1: 18.9: 17.3: 12.7 = 100. Genital double-somite (Fig. 5E) elongated, asymmetrical, with pointed process at each side on dorsal surface; that on right side larger than left counterpart and with patch of spinules placed nearby; genital operculum bumpy with two outgrowths, one of them bifid on right side. First and second free abdominal somites (Fig. 5C, D) with transverse hyaline frill both dorsally and ventrally. Anal somite shortest (Fig. 5F). Caudal rami (Fig. 5C, F), with 6 setae, symmetrical, about 1.63 times longer than wide (72 × 44 μm), with 4 transverse rows of spinules on ventral surface; caudal seta I lacking; seta II reduced; seta VII displaced to medial margin.

**Figure 6.**
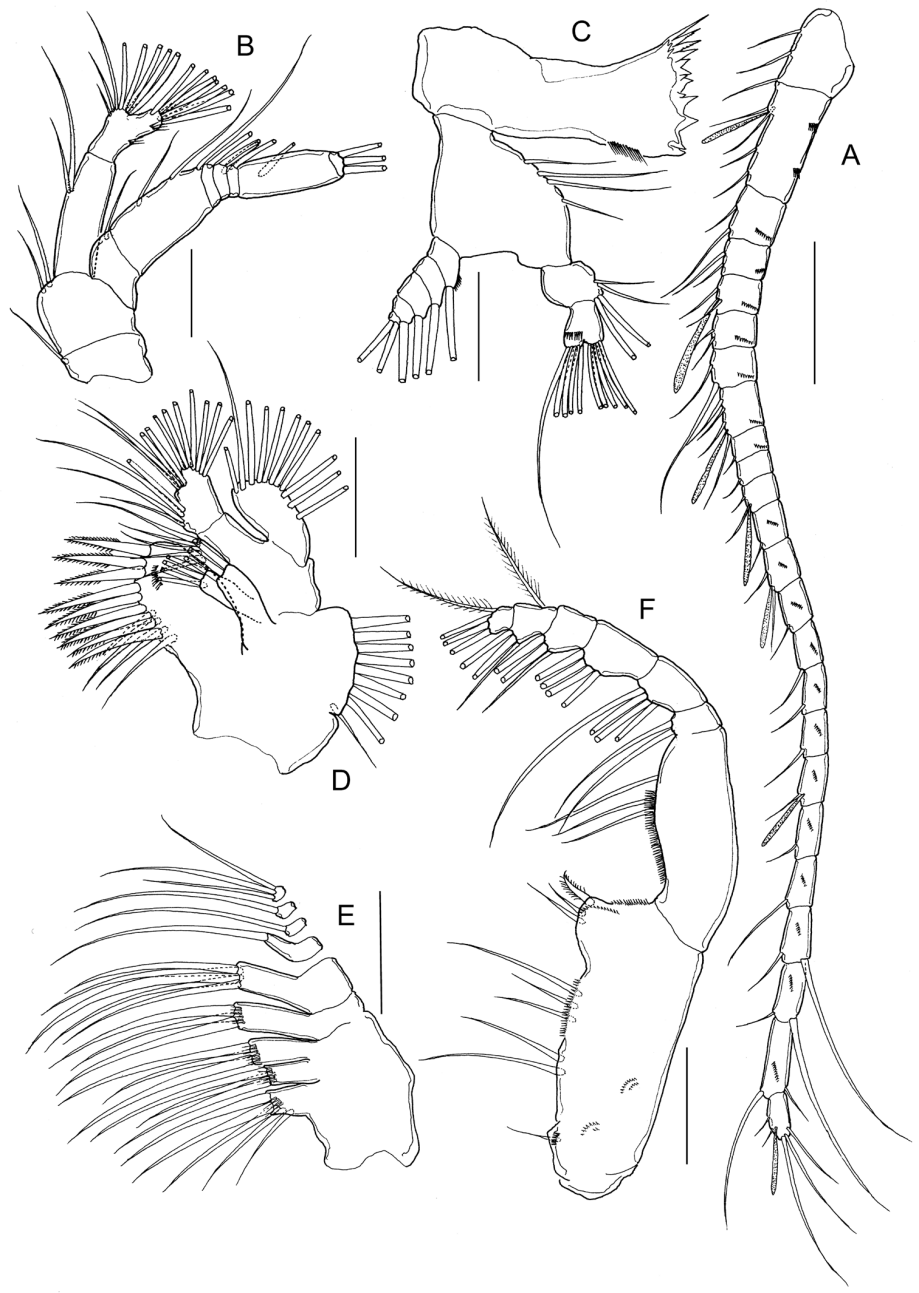
*Stephos projectus* sp. n., female paratype. **A** antennule **B** antenna **C** mandible **D** maxillule **E** maxilla **F** maxilliped. Scale bars = 50 µm.

Antennules (Fig. 6A) similar to preceding species except for not extending beyond distal margin of fifth pedigerous somite.

Antenna (Fig. 6B) similar to preceeding species except for presence of two transverse rows of spinules (instead of tiny serrated process plus spinule) on lateral margin of distal segment of endopod.

Mandible (Fig. 6C) similar to preceding species except for: (1) coxal gnathobase with straight row of moderately incised teeth; (2) outer margin of proximal segment of exopod with row of setules; and (3) distal segment of endopod with transverse row of spinules.

Maxillule (Fig. 6D) and maxilla (Fig. 6E) similar to preceding species except for presence of one additional seta on basal endite of maxilla.

Maxilliped (Fig. 6F) differing from *Stephos geojinensis* in presence of additional rows of tiny spinules on syncoxa.

**Figure 7.**
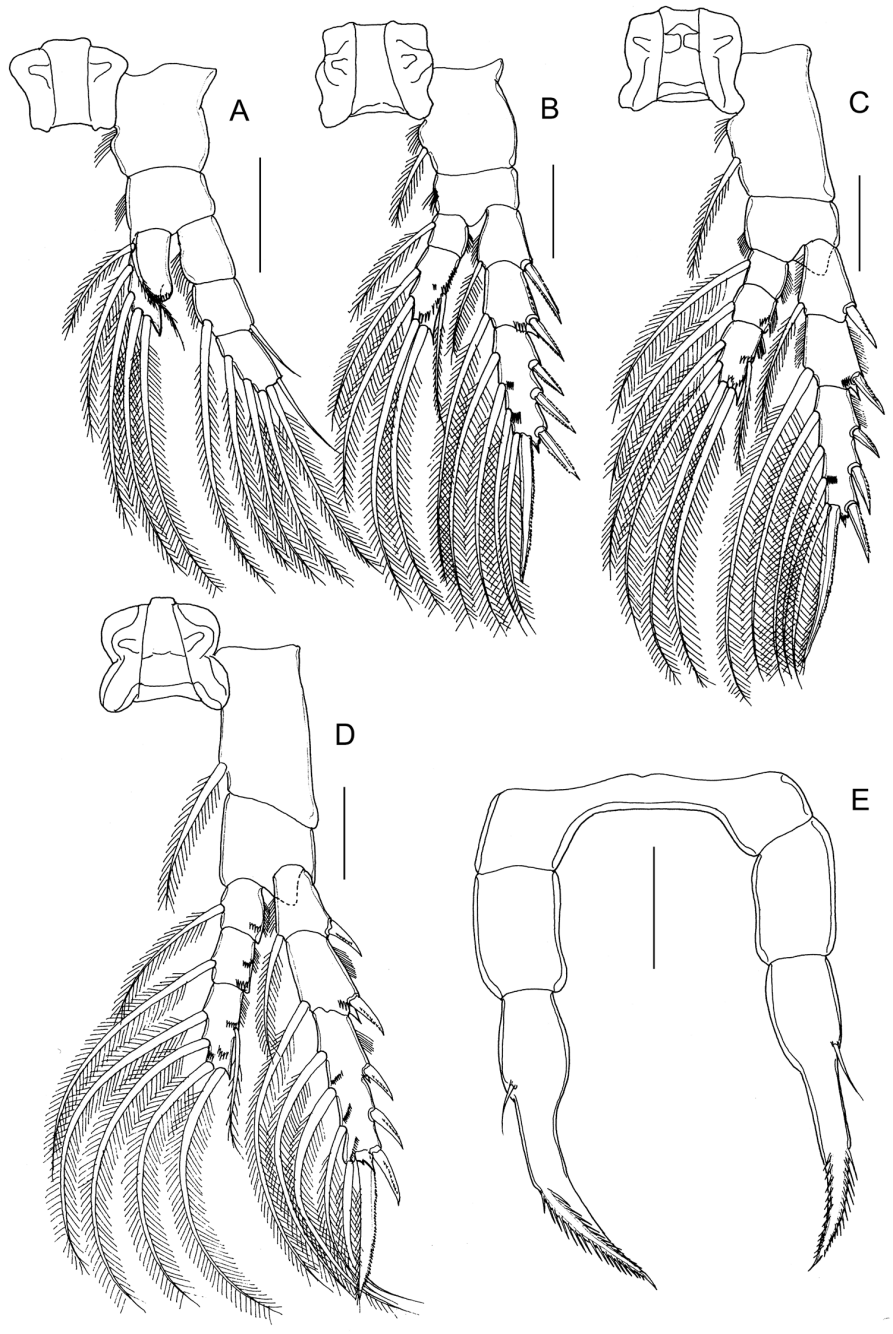
*Stephos projectus* sp. n., female paratype. **A** P1 **B** P2 **C** P3 **D** P4 **E** fifth legs. Scale bars = 50 µm.

P1 to P4 (Fig. 7A–D) with armature formula as in preceding species but with outer spine on second exopodal segment of P1 transformed into seta.

Fifth legs (Fig. 7E) symmetrical, uniramous, 3-segmented with proximal segment fused to intercoxal sclerite; second segment similar to *Stephos geojinensis* but shorter (1.72 times longer than wide; 31 × 18 μm); distal segment with a seta instead of spine on lateral margin, and with spinulation on terminal spine not so coarse.

##### Male.

**Figure 8.**
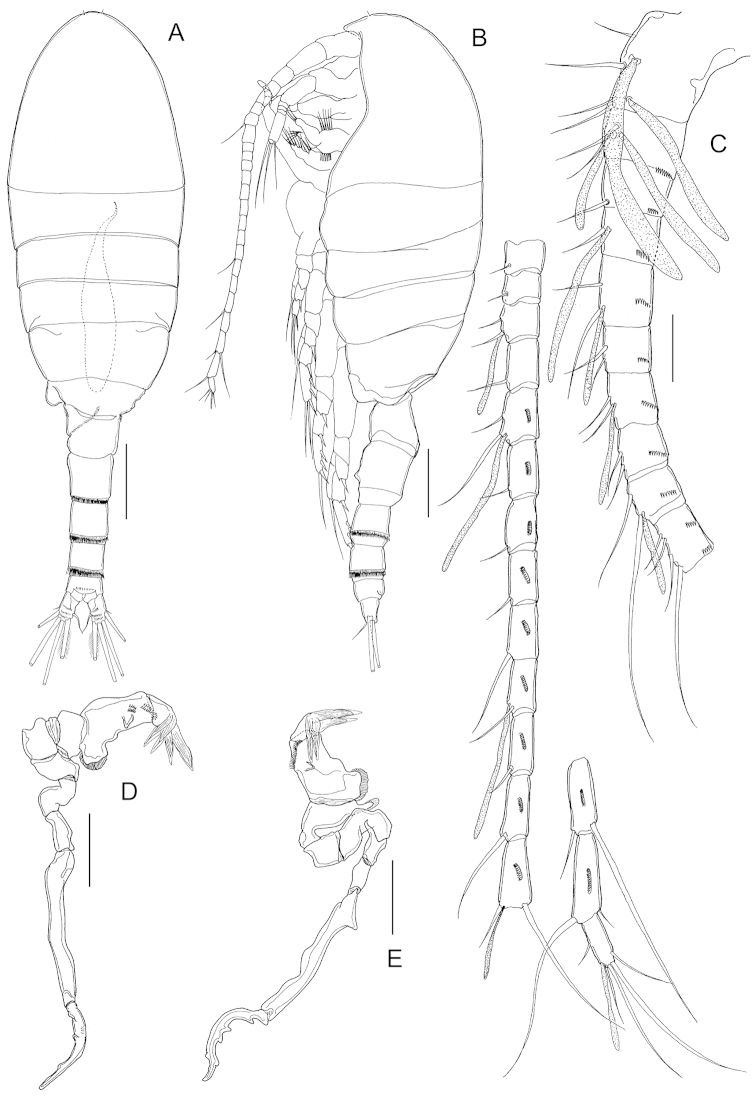
*Stephos projectus* sp. n., male paratype. **A** habitus, dorsal view **B** habitus, lateral view **C** antennule **D**, **E** fifth legs. Scale bars: **A, B** = 200 µm; **C–E** = 50 µm.

Body (Fig. 8A, B) robust, length 0.93 mm (mean 0.91±0.05, n=4) and similar to female in all major features except for last pedigerous somite, urosomal segmentation, armature of antennules and morphology of fifth legs. Fourth and fifth pedigerous somites, incompletely fused, latter asymmetrical with lateral lobe on left margin. Rostrum as in female. Prosome-urosome ratio 1.87:1. Urosome 5-segmented, comprising genital somite, three abdominal somites and anal somite; length ratio of urosomites as 23.7: 25.6: 22.6: 18.6: 9.5 = 100. Genital somite asymmetrical, with protruding lobe on left side and patch of tiny spinules proximally at each side. Abdominal somites with transverse hyaline frill both dorsally and ventrally. Anal somite shortest. Caudal rami similar to those of female.

Antennules (Fig. 8C) similar to preceding species except for not extending beyond distal margin of fifth pedigerous somite and for failure to express an aesthetasc on ancestral segments VI, VIII, composite X-XI, XX and XIII. In addition, the composite segment I-II displays 4 setae (vs. 3 in *Stephos geojinensis*).

Antenna, mandible, maxillule, maxilla, maxilliped and P1 to P4 similar to female.

Fifth legs (Fig. 8D, E) strongly asymmetrical, uniramous and filiform. Left leg 5-segmented; segments 3 and 4 each with rounded outgrowth on medial margin, outgrowth on segment 3 more slender and crowned with hyaline frill, that on segment 4 with ridged plate terminally; fourth segment with additional short conical outgrowth and longitudinal row of spinules; distal segment short, rounded, with 4 long hyaline lamellae on distal margin and patch of short spinules on posterior surface. Right leg 4-segmented; segment 3 elongate, straight and slender except for blunt triangular process proximally on lateral margin; fourth segment sickle-shaped with rounded tip; 4 rounded outgrowths along inner margin and single outgrowth subterminally on outer margin of segment.

##### Remarks.

*Stephos projectus* sp. n. falls within a group of species characterized by a 4-segmented right male P5 combined with a left leg in which the fourth segment is swollen (group III of Bradford-Grieve 1999). This group comprises 14 species from the Atlantic and the Indo-Pacific regions, namely: *Stephos boettgerschnackae* Kršinić, 2012, *Stephos canariensis* Boxshall, Stock & Sanchez, 1990, *Stephos cryptospinosus* Zagami, Campolimi & Costanzo, 2000, *Stephos deichmannae* Fleminger, 1957, *Stephos fultoni* T. Scott, 1898, *Stephos hastatus* Bradford-Grieve, 1999, *Stephos kurilensis* Kos, 1973, *Stephos lamellatus* G.O. Sars, 1902, *Stephos lucayensis* Fosshagen, 1970, *Stephos maculosus* Andronov, 1974, *Stephos minor* T. Scott, 1892, *Stephos robustus* Ohtsuka & Hiromi, 1987, *Stephos scotti* G.O. Sars, 1902, and *Stephos tropicus* Mori, 1942 (see Table 1 in Bradford-Grieve 1999; Zagami et al. 2000; Kršinić 2012). Only two of them, *Stephos hastatus* and *Stephos robustus*, share with the new species an asymmetrical female genital double-somite.

*Stephos projectus* sp. n. differs from *Stephos hastatus* in the following features: (1) presence of dorsolateral process at each side on the female genital double-somite (vs. processes absent, in *Stephos hastatus*); (2) fourth segment of male left P5 without strong spine (vs. strong spine present in *Stephos hastatus*); (3) fifth segment of male left P5 with 4 unequal long hyaline lamellae on distal margin (vs. two hyaline lamellae in *Stephos hastatus*); and (4) distal segment of male right P5 sickle-shaped (vs. segment bifid in *Stephos hastatus*). The new species can be easily differentiated from *Stephos robustus* based on the following features: (1) the presence of a dorsolateral pointed process at each side of the genital double-somite in female (vs. presence of a small mid-dorsal rounded process and not dorsolateral processes in *Stephos robustus*); (2) fifth segment of male left P5 with 4 long hyaline lamellae on distal margin (vs. 5 large spines of unequal length in *Stephos robustus*); and (3) fourth segment of male right leg sickle-shaped (vs. bifid in *Stephos robustus*).

The two new stephids described herein, *Stephos geojinensis* sp. n. and *Stephos projectus* sp. n., are easily differentiated based on the ornamentation of both the female genital double-somite and genital operculum; the morphology of the distal segment of the male right fifth leg; the presence/absence of a tiny pointed process on the distomedial angle of second segment of female P5; and the condition (seta or spine) of the lateral armature element on distal segment of female P5, among other features.

## Discussion

The genus *Stephos* shows many similarities in its morphological characteristics with its congener genera *Miostephos*, *Parastephos*, and *Speleohvarella*; however well, differs in the following characteristics: (1) the basal exite of the maxillule is present in *Stephos* and *Miostephos* (vs. absent in *Parastephos* and *Speleohvarella*), (2) the right P5 is 4-segmented in *Stephos* (vs. 5-segmented in *Parastephos* and 3-segmented in *Miostephos* and *Speleohvarella*); and (3) male right P5 is ending in unarmed claw and/or mitten-like segment (vs. claw is armed with spines along concave margin in *Parastephos* and reduced in *Miostephos* and *Speleohvarella*) (Boxshall and Halsey 2004; Kršinić 2005).

As an update we report that *Stephos* has 30 nominal species including the two described herein: *Stephos angulatus*, *Stephos antarcticum* Wolfenden, 1908, *Stephos articus* G.O. Sars, 1909, *Stephos boettgerschnackae*, *Stephos canariensis*, *Stephos cryptospinosus*, *Stephos deichmannae*, *Stephos geojinensis* sp. n., *Stephos exumensis* Fosshagen, 1970, *Stephos fultoni*, *Stephos gyrans* (Giesbrecht, 1893), *Stephos hastatus*, *Stephos kurilensis*, *Stephos lamellatus*, *Stephos longipes* Giesbrecht, 1902, *Stephos lucayensis*, *Stephos maculosus*, *Stephos margalefi* Riera, Vives & Gill, 1991, *Stephos marsalensis*, *Stephos minor*, *Stephos morii*, *Stephos pacificus*, *Stephos pentacanthos*, *Stephos projectus* sp. n., *Stephos robustus*, *Stephos rustadi*, *Stephos scotti*, *Stephos tropicus*, *Stephos tsuyazakiensis*, and *Stephos vivesi*. Six species of *Stephos* have so far been reported in Asian waters in particular East Asia: *Stephos pentacanthos* from China (Chen and Zhang 1965), *Stephos pacificus*, *Stephos robustus*, and *Stephos tsuyazakiensis* from Japan (Tanaka 1966; Ohtsuka and Hiromi 1987), and *Stephos geojinensis* sp. n. and *Stephos projectus* sp. n. from Korea (present study).

Members of *Stephos* are frequent in hyper- or epibenthic habitats of tropical to polar regions (Razouls et al. 2005–2014), and are occasionally recorded in caves (Boxshall et al. 1990; Riera et al. 1991; Jaume et al. 2008; Kršinić 2012). *Stephos geojinensis* sp. n. was collected at night using a plankton net and a light trap in near bottom shallow waters. Other stephids have also appeared in plankton samples collected at night in coastal waters (Kos 1972; Ohtsuka and Hiromi 1987; Costanzo et al. 2000; Zagami et al. 2000). We suggest that benthopelagic calanoids could undertake daily vertical migrations (Zagami et al. 2000) since many calanoids have a diel feeding rhythm with a maximum at night (Alldredge and King 1980; Head et al. 1985). The upward migratory behavior is a complex phenomenon related to factors such as feeding, reproduction, moulting, dispersal, and niche diversification (Alldredge and King 1980).

The second new species, *Stephos projectus* sp. n. was collected in the stagnant water flooding the burrows excavated byocypodid crabs in two intertidal mud flats. Cases of calanoid copepods associated with invertebrates have rarely been reported (Fosshagen 1970; Humes and Smith 1974; Moon and Soh 2014), whereas two epibenthic calanoid genera, *Placocalanus* Fosshagen, 1970 and *Boholina* Fosshagen, 1989, are known to burrow into the sediment temporally (Fosshagen 1970; Ohtsuka et al. 1996; Moon and Soh 2014).

## Supplementary Material

XML Treatment for Stephos geojinensis

XML Treatment for Stephos projectus
